# The 2018 Classification of Periodontal Diseases: An Observational Study on Inter-examiner Agreement among Undergraduate Students

**DOI:** 10.3290/j.ohpd.b5795649

**Published:** 2024-10-24

**Authors:** Majed K. Alshehri, Nujud Alamry, Shatha Subhi AlHarthi, Munerah Saleh BinShabaib, Ghaida Alkheraif, Mona Alzahrani, Raghad Bin Rubaian, Shikhah Binnjefan

**Affiliations:** a Assistant Professor/Periodontist, Preventive Dental Sciences Department, College of Dentistry, King Saud Bin Abdulaziz University for Health Sciences, Riyadh, Saudi Arabia; King Abdullah International Medical Research Center, Ministry of National Guard Health Affairs, Riyadh, Saudi Arabia. Research idea, study design.; b Assistant Professor/Periodontist, Department of Preventive Dental Sciences, College of Dentistry, Princess Nourah Bint Abdulrahman University, Riyadh, Saudi Arabia. Study design.; c Associate Professor/Periodontist, Department of Preventive Dental Sciences, College of Dentistry, Princess Nourah Bint Abdulrahman University, Riyadh, Saudi Arabia. Literature review.; d Associate Professor/Periodontist, Department of Preventive Dental Sciences, College of Dentistry, Princess Nourah Bint Abdulrahman University, Riyadh, Saudi Arabia. Wrote the manuscript.; e Teaching Assistant, Periodontology Division, Department of Preventive Dental Sciences, College of Dentistry, Princess Nourah Bint Abdulrahman University, Riyadh, Saudi Arabia. Data collection and analysis.; f Resident in Orthodontics and Dentofacial Orthopedics, Department of Preventive Dental Sciences, College of Dentistry, Princess Nourah Bint Abdulrahman University, Riyadh, Saudi Arabia. Orthodontics and Dentofacial Orthopedics Resident, Riyadh Second Health Cluster, Riyadh, Saudi Arabia. Data collection and analysis.; g General Dental Practitioner, Riyadh, Saudi Arabia. Data collection and analysis.; h Resident in Advanced Education in General Dentistry, Department of Preventive Dental Sciences, College of Dentistry, Princess Nourah Bint Abdulrahman University, Riyadh, Saudi Arabia. Department of Advanced Education in General Dentistry, National Guard Health Affairs, Riyadh, Saudi Arabia. Data collection and analysis.

**Keywords:** classification, diagnosis, inter-examiner agreement, periodontal disease, periodontitis, undergraduate

## Abstract

**Purpose:**

The objective of the present observational study was to assess the inter-examiner agreement for the diagnosis of periodontitis using the 2018 CPD among fourth and fifth year undergraduate students. It is hypothesised that there is no difference in the inter-examiner relaibility between fourth- and fifth-year undergraduate students regarding staging and grading periodontal disease using the 2018 Classification of Periodontal Diseases (CPD).

**Materials and Methods:**

All participants received training on the 2018 CPD scheme through a mandatory periodontics course conducted by a periodontist. Documentation for seven deidentified periodontitis patients, comprising medical history, dental history including tooth loss, intra-oral photographs and radiographs, periodontal charts reporting probing depth, plaque and bleeding on probing scores, furcation involvement and clinical attachment loss, was sent via e-mail to undergraduate students. The cases consisted of one sextant, and the participants were instructed to assume the sextant to be a true representation of the entire dentition. Power analysis was done on pilot data, and the level of significance was set at p<0.05.

**Results:**

The percentage of undergraduate students in the fourth and fifth year that correctly identified the stage of periodontitis according to the 2018 CPD ranged between 28% and 72% and 18.5% and 77.8%, respectively. The percentage of undergraduate students in the fourth and fifth year that correctly identified the grade of periodontitis ranged between 40% and 88% and 51.8% and 92.5%, respectively. The overall staging and grading ranged between 22.8% and 74.1%, and 45.66% and 87.4%, respectively. There was no statistically significant difference between fourth- and fifth-year undergraduate students with regards to assigning the correct diagnoses to case documentation in terms of either stage or grade.

**Conclusion:**

Fourth- and fifth-year undergraduate students demonstrated high inter-examiner agreement using the 2018 CPD.

Periodontitis is a prevalent and complex inflammatory disease affecting the supporting structures of teeth, leading to progressive attachment loss and bone resorption.^14,17^ A timely and accurate diagnosis and classification are crucial for effective treatment planning and management of periodontitis.^8,22^ In 2018, the American Academy of Periodontology and the European Federation of Periodontology introduced a revised classification system for periodontal and peri-implant diseases and conditions.^5^ This classification framework incorporates current understanding of the pathophysiology of periodontal diseases, emphasising staging based on severity and complexity of management, and grading based on the rate of disease progression and risk factors.^5^ Moreover, in this classification system,^5^ diseases of the periodontium were divided into three main categories: periodontal health and gingival diseases and conditions, periodontitis, and peri-implantitis. The terms “chronic” and “aggressive” periodontitis were eliminated due to the lack of evidence confirming these two to be distinct disease processes, and the previously separate types were grouped into one category; the re-classification was based on two vectors, staging and grading of periodontitis.^5,23^ In addition to aiding in diagnosis and communication, the staging and grading of periodontitis cases have eased the process of anticipating the disease’s progression rate, prognosis, and clearly identified the risk factors influencing the disease status.^16^

In an observational cross-sectional investigation, Abrahamiam et al^3^ assessed the inter-examiner reliability of postgraduate students towards classifying periodontitis using the 2018 classification. In that study,^3^ an online survey was sent to specialists in periodontology; the 174 respondents were requested to classify cases of periodontitis based on the 2018 classification. The results showed an inter-examiner agreement of approximately 69% and 82% for staging and grading of periodontitis, respectively.^3^ The authors concluded that the 2018 classification of periodontitis had a high inter-examiner reliability when used by specialists.^3^ However, it can be argued that postgraduate residents in periodontology possess considerably greater knowledge and clinical experience in the subject compared to undergraduate students. This factor likely contributes to the high inter-examiner reliability observed in the study by Abrahamiam et al.^3^ Similarly, Ravidà et al^18^ conducted a study to evaluate the inter-examiner agreement in staging and grading nine periodontitis cases among experienced periodontists. The results showed an interexaminer agreement of approximately 77% and 82% for staging and grading, respectively, among the periodontitis cases.^18^ From published, indexed studies,^3,18^ it is evident that the diagnostic efficacy of the new 2018 classification has exclusively been evaluated by experts (periodontists). A thorough review of relevant indexed literature reveals a dearth of studies that have assessed the diagnostic accuracy of the 2018 periodontitis classification protocol when used by undergraduate dental students (undergraduate students).

The objective of the present observational study was to assess the inter-examiner agreement in the diagnosis of periodontitis when fourth- and fifth-year undergraduate students used the 2018 Classification of Periodontal Diseases (CPD). The study hypothesis was that there would be no statistically significant difference in inter-examiner agreement.

## MATERIALS AND METHODS

### Ethical Approval

Ethical approval was obtained prior to the data collection process from the Institutional Review Board at Board at Princess Nourah bint Abdulrahman Univeristy (PNU), Riyadh, Saudi Arabia (HAP-01-R-059). An information sheet that explained the purpose and objectives of the present investigation was sent to all 4th- and 5th-year students at the the college of Dentistry, PNU, Riyadh Saudi Arabia. Participation was completely voluntary and the participants were aware that there would be no consequences for declining and/or withdrawal from the present study at any stage. Volunteers were requested to read and sign a written informed consent. All participants were invited to ask questions before and after signing the consent form.

### Study Design

This study employed an observational cross-sectional design to assess the diagnostic accuracy (inter-examiner agreement) of 4th- and 5th-year undergraduate students in staging and grading periodontitis using the 2018 CPD.

### Inclusion and Exclusion Criteria

The inclusion criteria were as follows: (a) undergraduate students enrolled in the fourth year at the College of Dentistry, PNU, Riyadh, Saudi Arabia; (b) undergraduate students enrolled in the final (fifth) year at the College of Dentistry, PNU, Riyadh, Saudi Arabia; (c) completion of training and course work for the diagnosis of periodontal diseases using the 2018 CPD;^5^ and (d) reading and signing the written informed consent. Refusal to participation was used as the sole exclusion criterion.

### Training of Participants

All participants received training in the 2018 CPD scheme during their third year of dental school through a mandatory periodontics course taught by a well-trained and experienced periodontist at the College of Dentistry, PNU, Riyadh, Saudi Arabia.

### Patient-based Cases and Expert’s Evaluation

**Fig 1:**
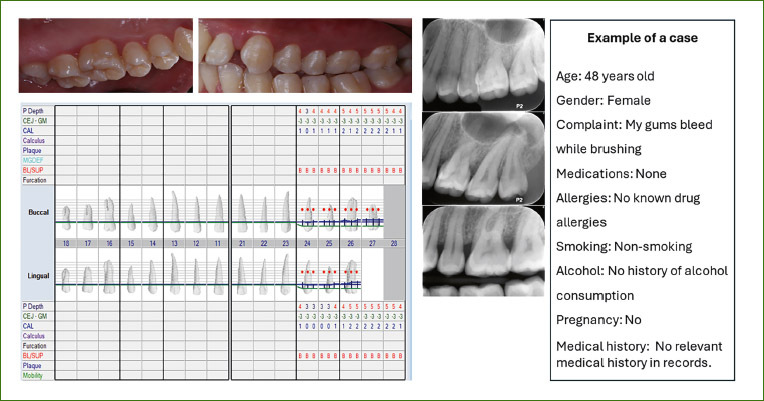
Case example.

Documentation for seven patients with a diagnosis of periodontits was randomly retrieved from the digital data pool of patients at the College of Dentistry, PNU, Riyadh, Saudi Arabia. These patients had provided written informed consent for the use of their anonymised data in the context of dental education, research and training. Patients with dental implants, periodontitis associated with systemic diseases such as diabetes mellitus, and acute periodontal inflammatory conditions were excluded.^1,12^ The cases comprised one sextant, and the participants were instructed to assume the sextant to be a true representation of the entire dentition. The documentation of each anonymous patient included: (a) medical history; (b) dental history including tooth loss; (c) intra-oral colour photographs; (d) intraoral radiographs of the sextant; (e) periodontal charts reporting periodontal probing depth, plaque and bleeding on gentle probing scores, furcation involvement and clinical attachment loss; and (f) tooth mobility. Prior to presentation of cases to undergraduate students, each case was assessed and diagnosed by a trained, calibrated and experienced periodontist (Kappa score 0.89) at the College of Dentistry, PNU, Riyadh, Saudi Arabia using the 2018 CPD.^5^ Figure 1 illustrates an example of a case sent to the undergraduate students.

### Evaluation of Cases

An e-mail was sent to all 4th- and 5th-year students at the College of Dentistry, PNU, Riyadh, Saudi Arabia, containing a link to the cases and their documentations. Only university-issued e-mail addresses were used for this communication. In the e-mail, students were instructed to maintain the confidentiality of patient-related information and were explicitly advised not to discuss or share the content with anyone. They were also reminded to exercise ethical and moral honesty when responding. Participants were requested to submit their responses within one week using a response link attached to the e-mail. Responses from all 4th- and 5th-year undergraduate students were then evaluated by a trained, experienced and calibrated faculty member (Kappa score 0.89; periodontist) of the College of Dentistry, PNU, Riyadh, Saudi Arabia, who was blinded to respondents’ educational (4th or 5th year) and personal details (e.g., name, age, and gender).

### Blinding of Examiner and Statistician

The periodontist evaluating responses submitted by the students was blinded to respondents’ educational and personal details. The statistician was also blinded to the parameters listed above.

### Power Analysis

Power analysis was done using a software program (G*Power software v. 3.1.9.7, Heinrich-Heine-Universität Düsseldorf, Düsseldorf, *Germany*)^13^ using data from a pilot investigation involving 10 participants each from the 4th and 5th year. The primary outcome variable was achieving at least 75% agreement (alpha and beta risks of 5% and 10%, respectivley) among 4th- and 5th-year undergraduate students regarding staging and grading of periodontitis using the CPD. It was estimated that at least 23 participants each from the 4th and 5th year are required to achieve 80% power for the study.

### Statistical Analysis

The data were coded and entered into SPSS Statistics version 28.0.1 (Chicago, IL, USA) and descriptive and analytical statistics were performed. Additionally, the percentages of agreement among the students were calculated for each year individually and collectively for both groups. Furthermore, the chi-squared test (statistical significance level of < 5%) was utilised to draw an inference regarding the presence of a statistically significant difference between the students based on their year of study (fourth and fifth).

## RESULTS

### Response to Invitation

The invitation was sent out to 52 female undergraduate students: 25 were 4th-year and 27 were 5th-year students. All invited undergraduate students agreed to participate in the present study and signed the informed consent form.

### Responses from Participants

The percentage of undergraduate students in the 4th and 5th year who correctly identified the stage of periodontitis according to the 2018 CPD ranged between 28% and 72% and 18.5% and 77.8%, respectively. The percentage of undergraduate students in the 4th and 5th year who correctly identified the grade of periodontitis according to the CPD 2018 ranged between 40% and 88% and 51.8% and 92.5%, respectivley. The overall staging and grading ranged between 22.8% and 74.1% and 45.7% and 87.4%. All participants demonstrated the least agreement on both the stage and grade (22.8% and 45.6%, respectively) of case 2. There was no statistically significant difference between 4th- and 5th-year dental students with regards to assigning the correct diagnoses to the case documentation in either staging or grading (Table 1).

**Table 1:** Frequency, percentages, and chi-squared tests for fourth- and fifth-year undergraduate students

Periodontitis cases	4th-year students (n = 25) (%)		5th-year students (n = 27) (%)		All participants (n = 52)		Pearson’s chi-squared test		p-value
	Staging	Grading	Staging	Grading	Staging	Grading	Staging	Grading	
Case 1 Stage I; Grade A	14 56%)	18 (72%)	13 (48.1%)	24 (88.8%)	51%	79.8%	0.571	0.123	0.16
Case 2 Stage II; Grade B	7 (28%)	10 (40%)	5 (18.5%)	14 (51.8%)	22.8%	45.6%	0.417	0.392	0.11
Case 3 Stage II; Grade B	16 (64%)	20 (80%)	18 (66.7%)	18 (66.7%)	64.6%	72.2%	0.84	0.279	0.11
Case 4 Stage III; Grade B	14 (56%)	17 (68%)	17 (62.9%)	23 (85.1%)	55.1%	76%	0.424	0.142	0.14
Case 5 Stage III; Grade C	14 (56%)	12 (48%)	18 (66.7%)	17 (62.9%)	60.8%	55.1%	0.430	0.278	0.2
Case 6 Stage IV; Grade C	18 (72%)	22 (88%)	21 (77.8%)	22 (81.4%)	74.1%	83.6%	0.631	0.515	0.17
Case 7 Stage IV; Grade C	16 (64%)	21 (84%)	21 (77.8%)	25 (92.5%)	70.3%	87.4%	0.427	0.333	0.15


## DISCUSSION

Implementation of any new classification system requires a period of learning, which may take a considerable amount of time to become familiar with and proficient in it. In this context, comprehensive training, appropriate implementation, supervision and consistent practice are essential to prevent misclassification, which otherwise may lead to incorrect diagnosis and faulty treatment plans.^6^ For over eight decades, the classification of periodontal diseases have repeatedly been revised,^15^ due to new case definitions based on aetiology (local and systemic), clinical expression and pathological changes. In other words, important new insights have been gained from cohort and epidemiological studies, basic research, and prospective studies evaluating environmental and systemic risk factors of periodontal diseases.^9,10,21,24^ This prompted the American Academy of Periodontology and the European Federation of Periodontology to formulate a new and upgraded classification for periodontal and peri-implant diseases. Since its acceptance in the year 2018, the new CPD has been used in several clinical periodontal investigations.^4,7,11,19^ In an editorial, Sanz et al^20^ explained how to use this classification system for staging and grading periodontal diseases and calculating tooth loss among patietns with stage III and IV periodontitis. Moreover, the limited number of studies^3,18^ that have assessed inter-examiner reliability regarding the new CPD have focused on specialists in periodontology. However, the authors of the present observational cross-sectional study recognise the paramount importance of educating 4th- and 5th-year undergraduate students about this classification system. Ensuring that these future dentists are well-versed in the new classification scheme by the time they graduate from dental instutions may better prepare them to serve as competent general dentists or to pursue advanced post-graduate programs in specialised fields such as periodontology, prosthodontics, and/or implant dentistry. With this objective in mind, the authors educated 4th- and 5th-year undergraduate students through lectures delivered by a trained periodontist, instructing them on how to diagnose periodontal diseases using the new 2018 classification system.^5^ In summary, results of the present study lead to acceptance of the null hypothesis, as no statistically significant differences in inter-examiner reliability were observed between 4th- and 5th-year students. This further indicates that any observed differences in the data are likely due to random chance rather than a true effect.

In a cross-sectional observational study, Abou-Arraj et al^2^ investigated the level of inter-examiner reliability among pre- and post-doctoral students from various educational stages and specialties in diagnosing and planning treatment for periodontal inflammatory conditions. In that study,^2^ second- and fourth-year undergraduate students and postgraduate residents in periodontology were included. Participants were presented ten cases of periodontitis with five choices of diagnosis.^2^ According to the results, respondents from the periodontology residency program demonstrated a higher level of inter-examiner agreement in terms of periodontal diagnosis compared with undergraduate students.^2^ Abou-Arraj et al^2^ concluded that undergraduate students showed lower inter-examiner agreement in diagnosing periodontal disases using the 2018 CPD. It is not surprising that in the study by Abou-Arraj et al,^2^ the level of training and education was higher among the postgraduate residents than among undergraduate students, which may have influenced the reported results. In contrast, in this study, all undergraduate students were trained and educated about the new 2018 CPD via a series of lectures by a well-trained and experienced periodontist. The current authors agree with Abou-Arraj et al,^2^ who stated that advanced training positively affected the level of agreement on diagnosis of periodontal diseases.

There are a number of limitations of the present study. First, the undergraduate students were required to diagnose the periodontal status of one sextant only. The primary rationale is that, despite being educated about the 2018 CPD, all undergraduate dental students had limited clinical experience. In clinical scenarios, the presentation of periodontal inflammation can vary across different quadrants, both clinically and radiographically. Given that undergraduate students are still in the process of developing their clinical and diagnostic skills, assigning them responsibility for diagnosing all jaw quadrants could overwhelm them and potentially lead to incorrect diagnoses. Moreover, the educational session conducted by the periodontist was carried out once before, alotting cases to participants. It is hypothesised that multiple educational sessions by trained faculty before the students perform and examination would help achieve a higher inter-examiner reliability, in contrast to the outcomes reported in the present investigation. Furthermore, the present study was exclusively conducted among undergraduate students. It is hypothesised that postgraduate residents in periodontics demonstrate a higher level of inter-examiner reliability, independent of the number of jaw sextants evaluated. Further longitudinal studies are needed to test these hypotheses. It is recommended that further training and curriculum revisions for undergraduate students regarding the updated classification of oral diseases including periodontitis should be formulated in the core syllabus. Such strategies may help improve diagnosis and treatment planning by general dentists as well as specialists, including periodontists.

## CONCLUSION

Fourth- and fifth-year undergraduate students demonstrated high inter-examiner agreement using the 2018 CPD.
